# Synergistic Effects of Curcumin and Piperine as Potent Acetylcholine and Amyloidogenic Inhibitors With Significant Neuroprotective Activity in SH-SY5Y Cells *via* Computational Molecular Modeling and *in vitro* Assay

**DOI:** 10.3389/fnagi.2019.00206

**Published:** 2019-08-27

**Authors:** Aimi Syamima Abdul Manap, Amelia Cheng Wei Tan, Weng Hhin Leong, Adeline Yoke Yin Chia, Shantini Vijayabalan, Aditya Arya, Eng Hwa Wong, Farzana Rizwan, Umesh Bindal, Shajan Koshy, Priya Madhavan

**Affiliations:** ^1^School of Biosciences, Faculty of Health and Sciences, Taylor’s University, Subang Jaya, Malaysia; ^2^School of Pharmacy, Faculty of Health and Sciences, Taylor’s University, Subang Jaya, Malaysia; ^3^School of Medicine, Faculty of Health and Sciences, Taylor’s University, Subang Jaya, Malaysia

**Keywords:** Alzheimer’s disease, amyloid beta, acetylcholinesterase, curcumin, piperine

## Abstract

Hallmarks of Alzheimer’s disease (AD) pathology include acetylcholine (ACh) deficiency and plaque deposition. Emerging studies suggest that acetylcholinesterase (AChE) may interact with amyloid β (Aβ) to promote aggregation of insoluble Aβ plaques in brains of patients. Current therapeutic options available for AD patients, such as AChE inhibitors, provide only symptomatic relief. In this study, we screened four natural compounds believed to harbor cognitive benefits—curcumin, piperine, bacoside A, and chebulinic acid. In the first section, preliminary screening through computational molecular docking simulations gauged the suitability of the compounds as novel AChE inhibitors. From here, only compounds that met the *in silico* selection criteria were selected for the second section through *in vitro* investigations, including AChE enzyme inhibition assay, 3-(4,5-dimenthylthiazol-2-yl)-2,5-dimethyltetrazolium bromide (MTT) assay, Thioflavin T (ThT) assay, and biochemical analysis *via* a neuronal cell line model. Of the four compounds screened, only curcumin (−9.6 kcal/mol) and piperine (−10.5 kcal/mol) showed favorable binding affinities and interactions towards AChE and were hence selected. *In vitro* AChE inhibition demonstrated that combination of curcumin and piperine showed greater AChE inhibition with an IC_50_ of 62.81 ± 0.01 μg/ml as compared to individual compounds, i.e., IC_50_ of curcumin at 134.5 ± 0.06 μg/ml and IC_50_ of piperine at 76.6 ± 0.08 μg/ml. In the SH-SY5Y cell model, this combination preserved cell viability up to 85%, indicating that the compounds protect against Aβ-induced neuronal damage (*p* < 0.01). Interestingly, our results also showed that curcumin and piperine achieved a synergistic effect at 35 μM with an synergism quotient (SQ) value of 1.824. Synergistic behavior indicates that the combination of these two compounds at lower concentrations may provide a better outcome than singularly used for Aβ proteins. Combined curcumin and piperine managed to inhibit aggregation (reduced ThT intensity at 0.432 a.u.; *p* < 0.01) as well as disaggregation (reduced ThT intensity at 0.532 a.u.; *p* < 0.01) of fibrillar Aβ42. Furthermore, combined curcumin and piperine reversed the Aβ-induced up-regulation of neuronal oxidative stress (*p* < 0.01). In conclusion, curcumin and piperine demonstrated promising neuroprotective effects, whereas bacoside A and chebulinic acid may not be suitable lead compounds. These results are hoped to advance the field of natural products research as potentially therapeutic and curative AD agents.

## Introduction

Alzheimer’s disease (AD) is the most common form of dementia, accounting for up to 60%–70% of cases worldwide. This neurodegenerative disorder mostly affects individuals aged 60 and above, with a projected prevalence of 100 million patients worldwide by the year 2050 (Dua et al., 2017). Despite the alarming figures, current therapeutic approaches available are merely symptomatic treatments that slow down cognitive decline with modest results; none of them are able to stop, prevent, or cure the disease (Galimberti and Scarpini, 2016). Three out of the four drugs available in the market, i.e., donepezil, galantamine, and rivastigmine, belong to the class of acetylcholinesterase (AChE) inhibitors. Based on their mechanism of action, targeting AChE pharmacologically for AD is reported to increase acetylcholine (ACh) concentration within the synapse, modulated through the binding of an inhibitor molecule to the catalytic action site (CAS) of the enzyme. Thus, with the decrease in ACh breakdown, it can compensate for the loss of functional neurons in early to mid-stages of the disease (Ali et al., 2015). As neurotransmission is reduced in the brains of AD patients due to the presence of neurotoxic amyloid plaques and fibrillary tangles, the increased levels of ACh are able to prolong cholinergic neurotransmissions and address cognitive symptoms such as learning and memory impairments (Kandimalla and Reddy, 2017). Unfortunately, as disease advances to the later stage with lesser intact neurons remaining, the drug action is likely insufficient to counteract the neurotransmission deficits. At this point, the downward spiral of cognitive function can no longer be kept in control (Mitchell et al., 2017).

Amyloid precursor protein (APP) discovered in healthy human brains is deemed to have a normal functional role. However, amyloid β (Aβ), a 39- to 43-amino-acid residue peptide produced from the proteolytic cleavage of APP, is believed to be the key fragment in propagating AD pathology (Castello and Soriano, 2014). The progressive deposition of Aβ aggregates is extensively credited to be fundamental to the primary development of neurodegenerative pathology through triggering a cascade of events such as neurotoxicity, oxidative damage, and inflammation that collectively contribute towards the progression of AD (Penke et al., 2017; Limbocker et al., 2018). In addition to plaque deposition, Aβ fibrils or soluble Aβ oligomers are intrinsically toxic to the cells by triggering the formation of reactive oxygen species (ROS), consequently attacking lipid membranes, which further facilitates lipid peroxidation while lowering the levels of first-line defense enzymes such as catalase and glutathione (Cheignon et al., 2018).

Therefore, emerging evidence of an area responsible for Aβ plaque aggregation activities within the AChE is promising, creating prospects of new treatment targets that potentially alter the course of disease progression, on top of managing the associated signs and symptoms. Identified as peripheral anionic site (PAS), an inhibitor compound at this site is expected to suppress plaque formation and deposition, hence interrupting with the downstream neuropathological cascade (Kračmarová et al., 2015). From here, the improved drug compound is proposed to interact simultaneously with both PAS and CAS of AChE. This offers an advantage compared to the drugs available at present; for example, galantamine binds to only CAS within the AChE and donepezil binds to either site, although it does so respectively, rather than both sites at once (Chierrito et al., 2017); hence, it is only marketed as a CAS inhibitor (Mehta et al., 2012). Furthermore, side effects such as nausea, vomiting, diarrhea, and dizziness have been commonly reported, which cause patients to have reduced adherence to their prescribed medications (Hansen et al., 2008). Thus, the search for a novel dual-site binding to AChE inhibitor is paramount to stop AD progression.

Of particular interest to researchers is in the area of natural products, in which the intrinsic bioactive compounds are known to possess various beneficial biological effects (Bui and Nguyen, 2017). This study investigates four natural compounds that are traditionally believed to offer cognitive benefits. These culinary herbs and medicinal remedies that are commonly used in Asia are curcumin from *Curcuma longa*, piperine from *Piper nigrum*, bacoside A from *Bacopa monniera*, and chebulinic acid from *Terminalia chebula*. The specific physiological benefits derived from these compounds are comprehensive, including epidemiological reports from improved cognitive functions in those who incorporate curcumin into their normal diet as opposed to those who do not (Ng et al., 2006). In addition, piperine attenuates memory impairment and neurodegeneration in cholinergic deficient rats (Chonpathompikunlert et al., 2010) while bacoside A is an iron chelator, where brain iron dyshomeostasis is strongly associated with AD pathology (Lane et al., 2018). Chebulinic acid, as well as the aforementioned three compounds exhibit anti-inflammatory and antioxidant effects, which have been reported to inhibit Aβ plaque formation and subsequently prevent Aβ-induced neurotoxicity (Afshari et al., 2016).

This study is divided into two parts: *in silico* screening followed by *in vitro* validation and further testing. Conducted through computational molecular docking simulations, this preliminary screening step gauges the suitability of the four compounds as novel AChE inhibitors. It investigates the binding affinities between the natural compounds and AChE receptor. Interactions of each ligand with key amino acids of receptor will also be studied to determine the abilities of the natural compounds in interacting with both CAS and PAS sites of AChE. From here, only the compounds that meet the *in silico* selection criteria would be selected for subsequent *in vitro* investigations *via* a neuronal cell line model, including AChE enzyme inhibition assay and reversal of oxidative damages. At the end of this study, ideal lead compounds, either individually or in combination, will be recommended for their neuroprotective effects. The results of this study is hoped to advance the field of natural products research as potential therapeutic and curative AD agents.

## Research Design

**Figure 1:**
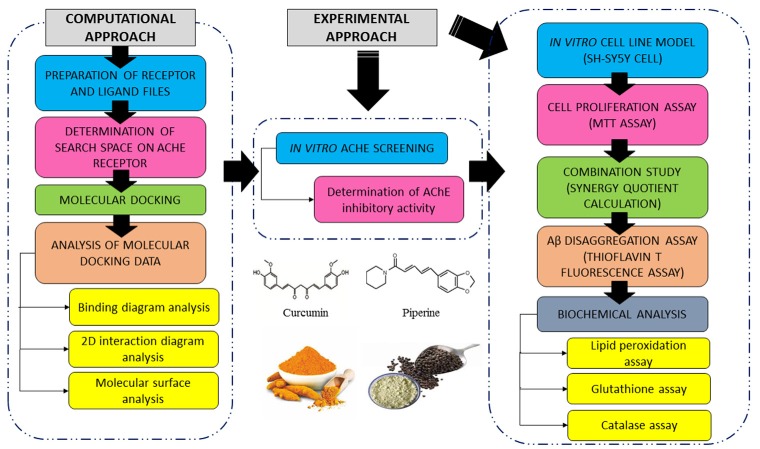
Summary of computational and experimental approach conducted in this study.

A summary of the research design is shown in Figure 1. In brief, four compounds were screened for their binding profiles with AChE receptor. The compounds that fulfilled the criteria of good binding affinity and key interactions with receptor were shortlisted for subsequent *in vitro* AChE inhibition activity where the compounds, individual and combined, were evaluated through maximal inhibition activity (IC_50_). We further evaluated the effects of selected compounds against Aβ-induced damages using the SH-SY5Y cell line. Details for each part of the methodology are further discussed in the following sections.

### *In Silico* AChE Screening

#### Preparation of Receptor and Ligand Files

The structure of AChE (PDB ID: 4EY6) with a resolution of 2.3983 Å was downloaded from Protein Data Bank in RCSB PDB format. Using Discovery Studio 2017 R2 (Dassault Systèmes BIOVIA, San Diego, CA, USA), the target structure was optimized by removing water molecules and all other ligands in the complex as they may affect the binding conformation and interactions. Among the ligands co-crystallized was galantamine, which is a type of FDA-approved AChE inhibitor drug. One of the identical chain in the homodimer structure was also deleted to minimize docking run time. Hydrogen atoms were then added and converted to pdbqt files.

The structures of ligands, curcumin (PubChem CID: 969516), piperine (PubChem CID: 638024), bacoside A (PubChem CID: 92033183), and chebulinic acid (PubChem CID: 250396) were downloaded from PubChem in SDF format. 3D format was chosen for curcumin and piperine. As bacoside A and chebulinic acid were only available in 2D formats, their 3D form was generated using OpenBabel software. The number of atoms was checked to ensure that no errors had occurred. All four ligands were converted to pdb format using Discovery Studio 2017 followed by conversion to pdbqt format using AutoDock Tools 1.5.6. Hydrogen atoms were added to the structures as well. Through root detection and setting of rotatable bonds, ligands were designated as flexible during the docking process. This is in line with the induced fit theory that generates a diverse set of ligand poses within the search space of receptor binding site (Clark et al., 2016).

### Determination of Search Space on AChE Receptor

**Figure 2:**
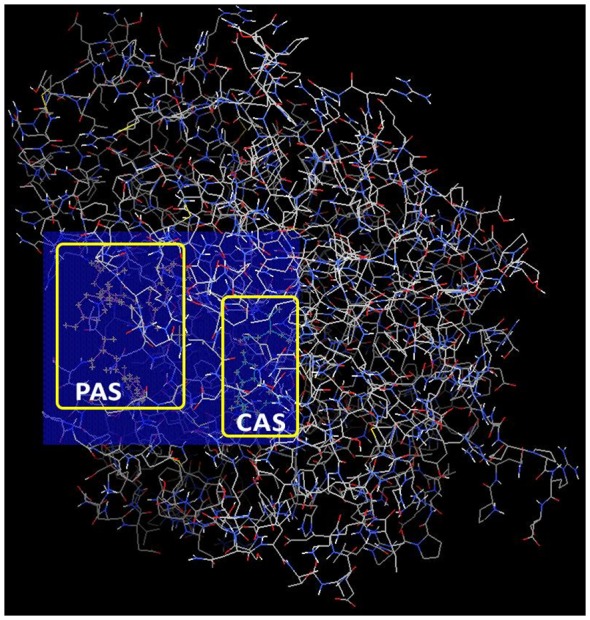
Relative position of AutoGrid box in acetylcholinesterase (AChE) encompassing both catalytic action site (CAS) and peripheral anionic site (PAS) docking sites.

The docking search site, where ligands would explore possible binding interactions with AChE, was determined using AutoDock Tools 1.5.6 (Morris et al., 2009). This search space (also known as AutoGrid) was positioned to encompass both CAS and PAS within the AChE gorge. The center for the 3D cuboidal AutoGrid box was set at (*x*: −12.3199, *y*: −42.071, *z*: 28.832) while the number of points in dimension for AutoGrid box was set at (*x*: 24, *y*: 20, *z*: 20). The relative position of AutoGrid in AChE is shown in Figure 2 below.

### Molecular Docking Using AutoDock Vina

Command prompt was used to perform the molecular docking runs. The command line for docking specified the locations of AutoDock Vina software, target receptor and ligand pdbqt files, configuration text file, as well as the desired location of output data. Each ligand-receptor pair under a specific AutoGrid box was subjected to 100 runs of docking, a sufficient number based on a docking system reviewed by Oferkin et al. (2015). In total, 400 runs were conducted for AChE target receptor at the end of this study. The resulting AutoDock Vina output files in pdbqt format recorded the poses generated while the text data listed binding energies for the corresponding poses (Trott and Olson, 2010).

#### Analysis of Molecular Docking Data

The two data obtained from molecular docking were the binding affinity in terms of binding energy (kcal/mol) and the visualization of binding conformation for each docking mode using PyMOL.

### Binding Diagram Analysis

From the poses generated, conformation of ligands with the least binding energy was extracted using PyMOL. The binding of each ligand to AChE target receptor was visualized subsequently.

### 2D Interaction Diagram

2D interaction diagram for the best pose of each ligand was captured using Discovery Studio 2017. The list of interactions was recorded and the bond diagrams between ligands were compared.

### Molecular Surface Analysis

The best pose of each ligand was subjected to hydrogen bonding and hydrophobicity plot analyses using Discovery Studio 2017.

### *In vitro* AChE Screening

#### Preparation of Compounds and Standard Drug

Following the findings from the molecular docking study, selected compounds and donepezil were dissolved in absolute dimethyl sulfoxide (DMSO) to make a 1,000 μg/ml stock solution and further diluted to desired concentrations.

#### Determination of AChE Inhibitory Activity

This assay was carried out following the protocol in the AChE assay kit (ABCAM, UK). This kit detects AChE inhibitory activity using DTNB to quantify the Thiocholine produced from the breakdown of Acetylthiocholine enabled by AChE. DTNB reacts with the sulfhydryl group (–SH) in Thiocholine and acts as a colored reagent (yellow) for colorimetry analysis. Absorbance readings were measured using a microplate reader at OD 410 nm. This assay can detect up to a minimum of 0.1 mU AChE in a 100-μL assay volume (1 mU/ml), which served as a reliable and sensitive test for this study.

#### Statistical Analysis

The absorbance readings were expressed as mean values of the triplicates, and a linear regression model showing the absorbance values of the standard solutions was plotted. The *R*^2^ value was obtained and evaluated. Next, the IC_50_ (the concentration required for 50% inhibition) values were calculated by constructing a non-linear regression graph between percentage inhibitions of selected compounds vs. concentration, using Graph Pad Prism software v.8.0.1 (GraphPad Software Inc., San Diego, CA, USA).

### *In vitro* Cell Line Screening

#### Cell Line and Cell Culture

Human neuroblastoma cells (SH-SY5Y) were purchased from ATCC, USA. The cell lines were maintained in DMEM (ATCC^®^ 30-2002™), supplemented with 10% FBS and 5% penicillin/streptomycin, and incubated in a humidified 5% CO_2_ 37°C incubator.

#### Cell Proliferation Assay

Cell proliferation assay was performed using the 3-(4,5-dimenthylthiazol-2-yl)-2,5-dimethyltetrazolium bromide (MTT) method. SH-SY5Y cells were seeded at 4 × 10^5^/ml in 96-well plates. When the cells reached 80% confluency, curcumin, piperine, and donepezil were added at different concentration gradients (curcumin: 2.5–100 μM; piperine and donepezil: 2.5–50 μM), followed by a 24-h incubation. Stock solution of MTT (Sigma; 5 mg/ml) was added into each well and incubated at 37°C in a CO_2_ incubator for 3 h. MTT medium was then carefully aspirated from the wells, and the formazan dye was eluted using DMSO. Absorbance was measured using SPECTROstar Nano microplate reader (BMG Labtech, Cary, NC, USA) at a wavelength of 570 nm and formazan product was measured as an index of cell proliferation. Cell viability percentage was calculated and expressed relative to the value in the vehicle-treated control culture. Effective concentration (EC) of each individual compound was optimized and selected for further experiments.

#### Combination Study

The concentration of compounds prepared for testing of the combinatorial effect was in 2-fold dilutions from the final concentration. These compounds were added in a ratio of 1:1, using the checkerboard dilution method (Chang et al., 1995; Su Wei Poh et al., 2015).

### Synergy Quotient Calculation for Synergism

The synergism quotient (SQ) was calculated by subtracting baseline values from all treatments and then dividing effects of combined treatments by the sum of individual treatments. An SQ greater than 1.0 indicates a synergism for a given measured response.

### Aβ Disaggregation Assay

#### Aβ Preparation

Synthetic Aβ42 fibril was prepared as described previously with slight modifications (Tycko, 2018). In brief, the Aβ42 peptide was dissolved in 1,1,1,3,3,3-hexafluoro-2-propanol (HFIP, Sigma) at 1 mg/ml and aliquoted in Eppendorf tubes. The HFIP was allowed to evaporate in the fume hood, and the resulting clear peptide film was dried under vacuum overnight. The traces of HFIP were removed under vacuum (Speed Vac; Thermo Fisher Scientific, Waltham, MA, USA) on the next day and resuspended in DMSO to a concentration of 5 mM. To form fibrillar conditions, the peptide was diluted to a final concentration of 100 μM with 10 mM of acid hydrochloric (HCI) solution and incubated at 4°C for 24 h.

#### Thioflavin T (ThT) Fluorescence Assay

To test the inhibition and disaggregation effect of curcumin and piperine on Aβ fibrils, 25 μM Aβ42 fibril was incubated with 5 μM Thioflavin T (ThT) solution. Fluorescence intensity was monitored at an excitation wavelength of 450 nm and an emission wavelength of 482 nm using a spectrometer.

### Biochemical Analysis

#### Measurement of Malondialdehyde (MDA), Glutathione, and Catalase

The malondialdehyde (MDA) level was measured using Lipid Peroxidation Kit (Abcam, UK). In this assay, the MDA in the sample was allowed to react with Thiobarbituric Acid (TBA) to generate the MDA-TBA adduct. The MDA-TBA adduct was quantified colorimetrically at a wavelength of 532 nm. Reduced glutathione (GSH) level was measured using Glutathione peroxidase (GPx) Assay Kit (Abcam, UK). In this assay, GPx reduces cumene hydroperoxide while oxidizing GSH to oxidize glutathione (GSSG). The generated GSSG is reduced to GSH with consumption of NADPH by glutathione reductase (GR). The decreased NADPH is proportional to GPx activity, which the absorbance was measured at 340 nm in this study. Catalase activity was determined with catalase assay kit (Abcam, UK). In the assay, catalase first reacts with hydrogen peroxide (H_2_O_2_) to produce water and oxygen, and the unconverted H_2_O_2_ reacts with OxiRed™ probe to produce a product, which was measured colometrically at 570 nm.

#### Statistical Analysis

All data were expressed as mean ± SEM (standard error of the mean). Data collected were analyzed using one-way ANOVA *via* a statistical software GraphPad Prism version 8.0 (GraphPad Software Inc., San Diego, CA, USA), where *p < 0.05 and **p ≤ 0.01.

## Results

### Computational Approach

#### Analysis of Binding Affinity and Binding Energy

Molecular docking of the four ligands to AChE was carried out using AutoDock Vina 1.1.2. Following 100 docking runs for each ligand and data sorting, the results for the best binding conformation of each ligand to AChE are tabulated in Table 1. From Table 1, negative binding energies were recorded for all four ligands, indicating that they were generally favorable and have a stable binding to AChE receptor. Based on the binding affinities of the ligands, we postulate that the binding between piperine to AChE receptor is the most stable, followed by curcumin, chebulinic acid and bacoside A.

**Table 1:** Binding energies and interactions observed between the top pose of each ligand with acetylcholinesterase (AChE) receptor.

Ligand	Binding affinity (kcal/mol)	Interaction(s) with AChE sites	
		CAS	PAS
Curcumin	−9.6	√	√
Piperine	−10.5	√	√
Bacoside A	−3.2		√
Chebulinic acid	−6.7		√

#### 2D Interaction Diagram Analysis

**Figure 3:**
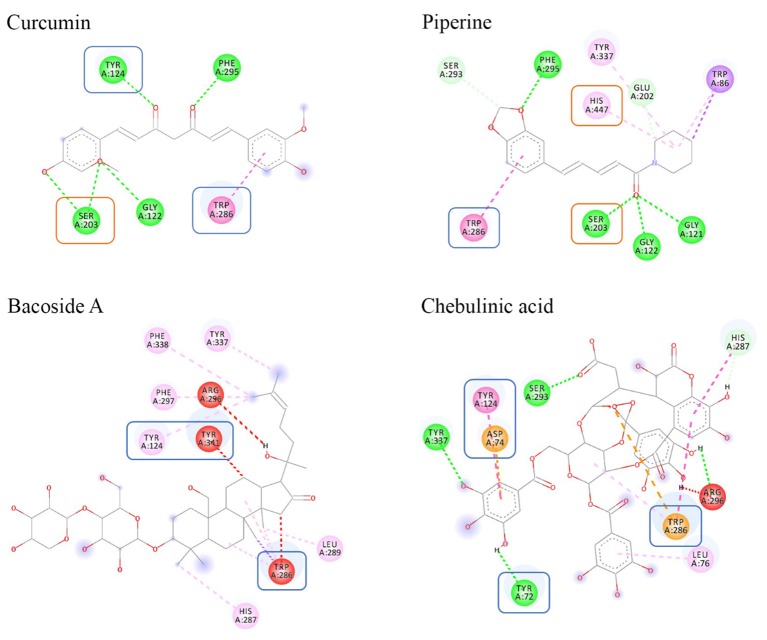
Binding interactions of ligands to AChE. Amino acids in orange box represent CAS residues, blue box for PAS, while the remaining amino acids are residues that were involved in stabilizing the binding within the AChE gorge.

**Figure 4:**
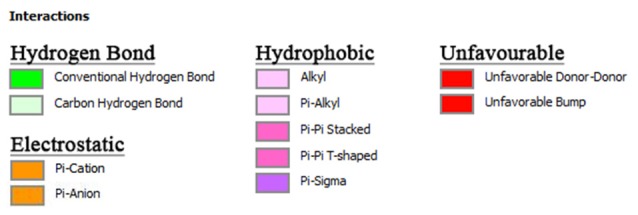
Color coding of non-covalent interactions linking ligand–receptor pairs. The interaction can be categorized into five groups, namely, hydrogen bonds, electrostatic, hydrophobic, and unfavorable linkage.

The 2D interaction diagrams for each docked ligand within AChE were generated using Discovery Studios 2017 (Figures 3, 4). We analyzed and compared the interactions, which provided valuable information on the docking profiles of each ligand. Detailed information of each bond formed is included in Supplementary Table S1.

In this study, the search space of AChE was defined to encompass two distinct binding sites, CAS and PAS. The CAS consisted of amino acids SER203, HIS447, and GLU334 whereas the PAS consisted of amino acids TYR72, ASP74, TYR124, TRP286, and TYR341. Based on Figure 3, only curcumin and piperine were able to bind to both CAS and PAS sites simultaneously. It showed that curcumin bound to SER203 in the CAS and to TYR124 and TRP286 in the PAS, while piperine bound to SER203 and HIS447 in the CAS and to TRP286 in the PAS. Absence of CAS binding was noted in both bacoside A and chebulinic acid. Additionally, unfavorable binding was seen between bacoside A and chebulinic acid to AChE, as denoted by the presence of red lines.

#### Molecular Surface Analysis

**Figure 5:**
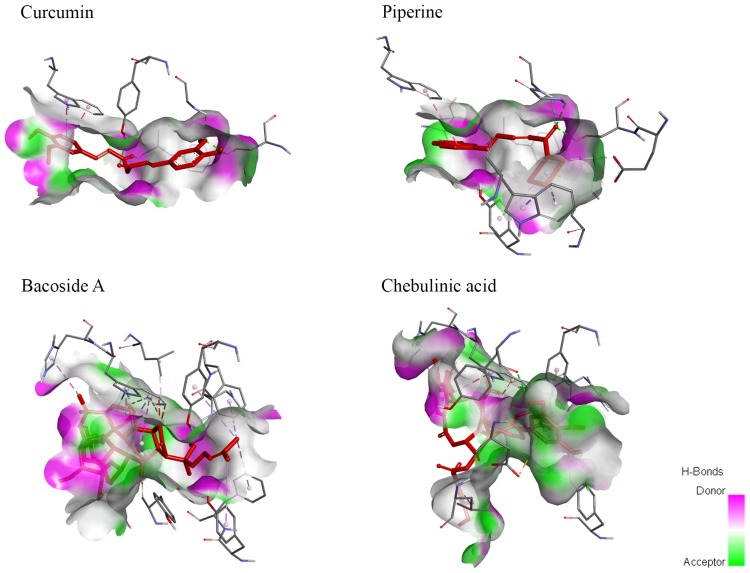
Hydrogen bond molecular surface mapped the locations of hydrogen bonds in ligands when bound to AChE. Curcumin and piperine demonstrated a high number of hydrogen bond donors and hydrogen bond acceptors. Meanwhile, bacoside A showed a higher number of hydrogen bond donors while chebulinic acid presented with a higher number of hydrogen bond acceptors.

**Figure 6:**
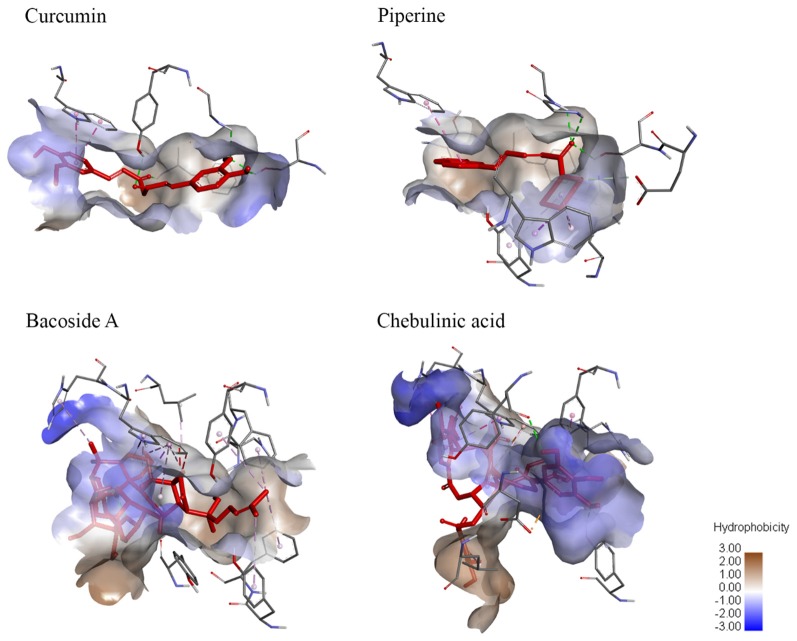
Degree of hydrophobicity on the molecular surface of ligands when bound to AChE. Curcumin and piperine both have similar sizes of areas with low hydrophobicity and areas of low hydrophilicity. Meanwhile, bacoside A has a larger area of hydrophobicity while chebulinic acid has a smaller area of hydrophobicity.

Molecular surface analysis is divided into two sections—hydrogen bonding and degree of hydrophobicity. Hydrogen bonding provides further understanding on the differences in binding affinity while the degree of hydrophobicity is important in the pharmacokinetic properties of a drug. Both hydrogen bonding and hydrophobicity interaction diagrams between ligands and AChE were generated using Discovery Studios 2017 and demonstrated in Figures 5, 6.

The number of hydrogen bonds can affect the binding affinity of ligand towards receptor. Based on Supplementary Table S1, CAS and PAS of AChE consist of mainly hydrogen bond donors. As hydrogen bonding requires a hydrogen bond donor and an acceptor to form the bond, chebulinic acid should have the highest binding affinity, whereas bacoside A should have the lowest binding affinity. From the hydrophobicity results, it is indicated that bacoside A would have the highest value of hydrophobicity (log *P*) while chebulinic acid has the lowest log *P*-value.

Based on our computational results, only curcumin and piperine were selected for subsequent *in vitro* studies.

### Experimental Approach

#### *In vitro* AChE Inhibition Assay

A low IC_50_ value suggests better efficacy and lower potency of drug required for enzyme inhibition. Out of all the test compounds studied, combined curcumin and piperine (IC_50_: 62.81 ± 0.01) exhibited comparable enzymatic inhibitory effect to donepezil (IC_50_: 54.79 ± 0.01; Table 2). Likewise, the IC_50_ values for combined curcumin and piperine were much higher than the individual curcumin (134.5 ± 0.06) and piperine (76.6 ± 0.08).

**Table 2:** Percentage of AChE inhibition and IC_50_ results for selected test compounds.

Test compounds	% Inhibition					IC_50_ ± SEM (μg/ml)
	25 μg/ml	50 μg/ml	100 μg/ml	250 μg/ml	500 μg/ml	
Donepezil	63.66	71.43	90.22	90.30	90.30	54.79 ± 0.01
Curcumin	7.14	18.01	31.68	51.86	71.43	134.5 ± 0.06
Piperine	21.97	48.06	54.27	75.08	80.98	76.6 ± 0.08
Curcumin + Piperine	12.5	20.26	90.37	90.84	91.23	62.81 ± 0.01

#### *In vitro* Cell Line Screening

##### Curcumin and Piperine Induced Dose-Dependent Increase in Cell Proliferation in SH-SY5Y Cells

**Figure 7:**
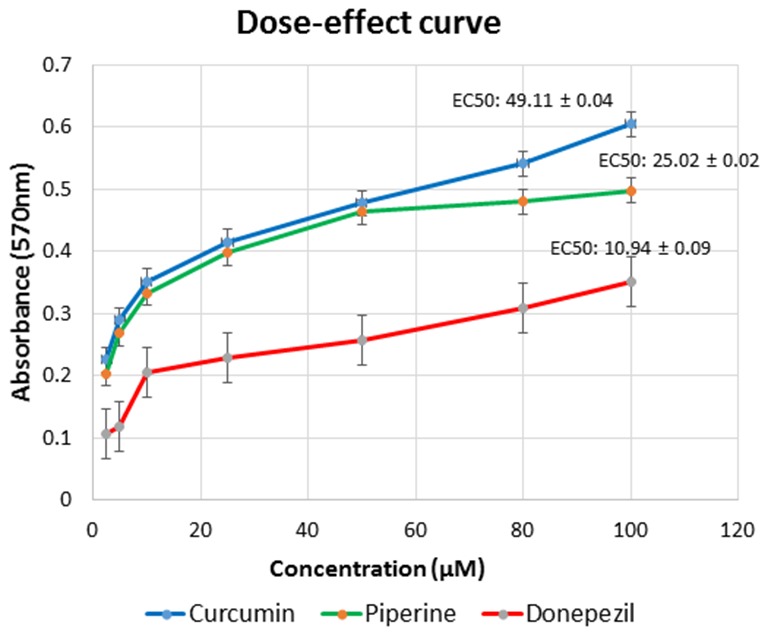
Dose–response relationships for curcumin, piperine, and donepezil used singularly in various concentration gradients from 2.5 μM to 100 μM. Each curve represents the increase in cell viability as a function of the dose. Effective concentration (EC) was calculated using GraphPad Prism.

In our *in vitro* cell line model, we observed the degeneration effects of Aβ in cultured SH-SY5Y cells that served as a neuronal cell model. Our dose–effect curve result showed that curcumin achieved EC_50_ at 49.11 μM, piperine at 25.02 μM, and donepezil at lower concentration, which was at 10.94 μM after 24 h incubation in SH-SY5Y cells (Figure 7).

##### Activity of Combined Curcumin and Piperine in the Combination Treatment

**Figure 8:**
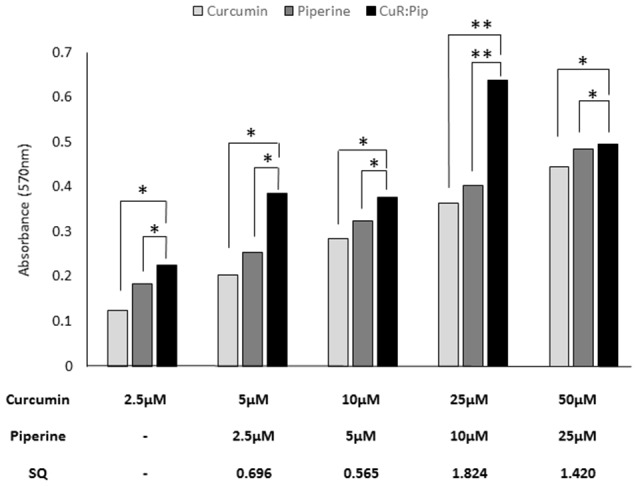
Effect of varying concentrations of curcumin and piperine used in combination in SH-SY5Y cells proliferation after 24 h. Synergism quotient (SQ) values were calculated to determine if there was any synergistic activity in the treatment. The results presented are averages of three independent experiments each done in triplicate and expressed as the mean ± SEM. *p < 0.05, **p < 0.01, one-way ANOVA with Dunnett’s test compared to combined curcumin and piperine group (CuR: Pip).

For the combination study, synergistic activity was observed between 25 and 50 μM of curcumin and 10–25 μM of piperine (total dose, 35 μM), calculated based on the SQ calculation (Figure 8). The maximum synergistic activity was observed at 25 μM of curcumin and 10 μM of piperine, with an SQ of 1.824. In this study, higher concentration of curcumin (25–50 μM) with piperine (10–25 μM) was shown to induce a synergistic activity, while an antagonistic activity was observed with lower concentrations of curcumin (2.5–10 μM) and piperine (2.5–5 μM). This could be because both curcumin and piperine exert activity at higher concentrations, singularly.

##### Combined Curcumin and Piperine Against Aβ-Induced Cytotoxicity

**Figure 9:**
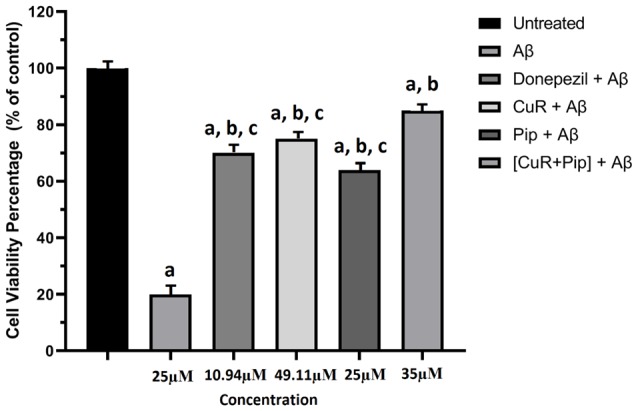
Effects of amyloid β (Aβ), Donepezil + Aβ, curcumin + Aβ (CuR + Aβ), piperine + Aβ (Pip + Aβ), and combined curcumin and piperine + Aβ [(CuR + Pip) + Aβ] in SH-SY5Y cell proliferation after 24 h treatment. The cell viability percentage was calculated relative to the untreated group and expressed as the mean ± SEM. ^a^*p* < 0.05 compared to untreated cells, ^b^*p* < 0.01 compared to the Aβ group, ^c^*p* < 0.01 compared to the (CuR: Pip) + Aβ group (one-way ANOVA followed by Tukey’s test).

The cells treated with 25 μM of Aβ42 in the absence or presence of all test compounds and their viabilities were measured using the MTT assay. In the absence of test compound, 25 μM Aβ42 exhibited a substantial toxic effect, which reduced cell viability to 20% (Figure 9). However, pre-incubation of all test compound with Aβ42 (25 μM) presented a pronounced decrease in cell toxicity, where cell viability was effectively restored up to 85%, demonstrating a distinct effect of compound upon Aβ42 cytotoxicity. We also compared the effect of individual compound and combined compound against cytotoxicity. The combination of curcumin and piperine at 35 μM significantly preserved cell viability (85%) as compared to individual compounds (curcumin: 75%, piperine: 65%) at *p* < 0.01.

#### Aβ Disaggregation Assay

##### Combined Curcumin and Piperine Inhibit and Disaggregate Fibril Formation

**Figure 10:**
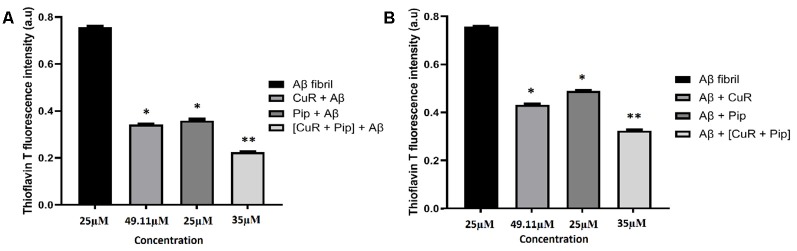
Thioflavin T (ThT) fluorescence assay of SH-SY5Y cells treated with Aβ, Donepezil + Aβ, curcumin + Aβ (CuR + Aβ), piperine + AB (Pip + Aβ), and combined curcumin and piperine + AB [(CuR + Pip) + Aβ] on **(A)** inhibition and **(B)** disaggregation of preformed Aβ fibrils (*n* = 3, *p < 0.05, **p < 0.01 compared to the Aβ group, one-way ANOVA followed by Tukey’s test).

From our findings, ThT analysis demonstrated that in both cases, all compounds clearly inhibited fibrillation of Aβ42 as shown in Figure 10A and disaggregated the fibrils as shown in Figure 10B, as reflected in the lower intensity of the ThT fluorescence. Interestingly, more pronounced inhibition (reduced ThT intensity at 0.432 a.u. compared to the Aβ group) and disaggregation (reduced ThT intensity at 0.532 a.u. compared to Aβ group) of Aβ42 fibrillation were observed with the combined curcumin and piperine.

#### Biochemical Analysis

##### Combined Curcumin and Piperine Attenuates Aβ-Induced Oxidative Damage

**Figure 11:**
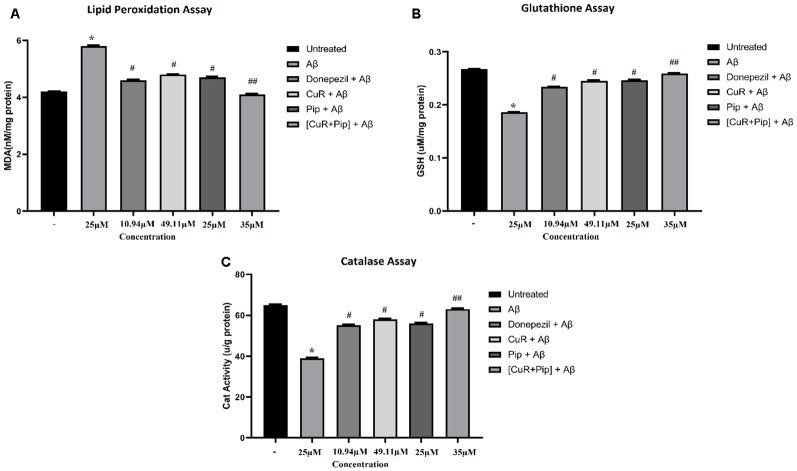
Biochemical assay of SH-SY5Y cells treated with Aβ, Donepezil + Aβ, curcumin + Aβ (CuR + Aβ), piperine + Aβ (Pip + Aβ), and combined curcumin and piperine + Aβ [(CuR + Pip) + Aβ]. **(A)** Lipid peroxidation assay, **(B)** glutathione assay, **(C)** catalase assay. Values were presented as mean ± SD, from three independent experiments. *p < 0.01, compared to untreated group. ^#^*p* < 0.05, ^##^*p* < 0.01, compared to the Aβ group (one-way ANOVA followed by Tukey’s test). MDA, malondialdehyde; GSH, glutathione; Cat, catalase.

As shown in Figure 11, upon pre-incubation of Aβ42 at 25 μM in cultured SH-SY5Y cells for 24 h, Aβ42 caused a substantial increase in the level of lipid peroxidation end product: (a) MDA level (increased 1.6 nM/mg protein in MDA level compared to untreated group; *p* < 0.01); (b) with an obvious decrease in the level of glutathione (reduced 0.081 μM/mg protein in glutathione level compared to untreated group; *p* < 0.01); and (c) catalase enzyme (reduced 26 μ/g protein in catalase level compared to untreated group; *p* < 0.01). For the groups that received treatment of all compounds singularly including donepezil, curcumin, and piperine followed by addition of Aβ, results showed a significant reduction (*p* < 0.05) in the level of MDA (total reduction: donepezil + Aβ: 1.2 nM/mg protein, CuR + Aβ: 1.0 nM/mg protein, and Pip + Aβ: 1.1 nM/mg protein compared to the Aβ group) while significant elevation (*p* < 0.05) in the level of antioxidant enzyme, glutathione (total elevation: donepezil + Aβ: 0.048 μM/mg protein, CuR + Aβ: 0.059 μM/mg protein, and Pip + Aβ: 0.06 μM/mg protein compared to the Aβ group), and catalase level (total elevation: donepezil + Aβ: 16 μ/g protein, CuR + Aβ: 19 μ/g protein, and Pip + Aβ: 17 μ/g protein to the Aβ group). However, combination of curcumin and piperine at 35 μM exerted greater effects (reduced 1.7 nM/mg protein in MDA level, increased 0.073 μM/mg protein in glutathione level, and increased 24 μ/g protein in catalase level compared to the Aβ group) than compounds used singularly at *p* < 0.01.

## Discussion

With unparalleled bioactive components and structural diversity, natural products remain the most pivotal source of novel compounds for therapeutic drugs development (Yuan et al., 2016). Considering that Asia is home to a plethora of flora and the wide use of medicinal herbs and culinary spices across the continent, elucidating the bioactivity of these phytochemicals would give a better understanding of its mechanism of action and potential adaptation into pharmaceutical drugs (Jantan et al., 2015). Molecular docking was conducted on curcumin, piperine, bacoside A, and chebulinic acid, all of which are traditionally believed to harbor nootropic benefits (Chonpathompikunlert et al., 2010; Goozee et al., 2016; Nemetchek et al., 2017; Uddin et al., 2019). In this study, *in silico* docking was first employed as an efficient and cost-saving method for the initial filtering of lead candidates in drug discovery for AD. Through simulations, the binding affinities and types of interactions of each ligand within the binding pocket of AChE receptor were identified. Following screening and selection of compounds, *in vitro* studies were then carried out for validation and further testing.

Binding affinity, also termed binding energy, is the energy released or consumed when a ligand binds to its receptor (Purich, 2010). If the binding occurs spontaneously and is thus favorable, the overall energy of the complex would be reduced, as denoted by a negative score. Conversely, unfavorable binding consumes energy and is given a penalty of positive score. On an arbitrary scale, the more negative is the binding energy, the more stable the ligand-receptor complex is, and vice versa (Du et al., 2016). As summarized in Table 1, although all ligands recorded negative energies, curcumin and piperine at −9.6 and −10.5 kcal/mol were evidently the stronger ligands that bound to AChE receptor, as compared to bacoside A and chebulinic acid at −3.2 and −6.7 kcal/mol, respectively. In curcumin and piperine, the lower binding energies were the results of hydrogen bonds, electrostatic charges, and hydrophobic interactions, forming a stable docked complex. As for the small differences noted, it can be explained through the number of bonds formed between ligand and receptor. Piperine formed six hydrogen bonds and five hydrophobic interactions with AChE, whereas curcumin only formed four hydrogen bonds and one hydrophobic bond with AChE, hence the greater affinity in piperine. However, in bacoside A and chebulinic acid, although similar non-covalent interactions were observed, the overall affinity towards AChE receptor was reduced by unfavorable bond(s), indicating repulsion forces that destabilized the complex. Considering that in the *in vivo* state, with AChE inhibitors being in competition with endogenous ACh substrate for binding to the receptor, we believe that AChE would preferentially bind to the ligand that has a more favorable affinity towards it. Hence, it is imperative that potential AChE inhibitors exhibit greater binding stability to maximize on its enzymatic inhibition and plaque aggregation prevention properties.

Binding affinities were also determined by the molecular size and structure of the ligands (Atkovska et al., 2014). Based on molecular weights (MW), both bacoside A (768.982 g/mol) and chebulinic acid (956.68 g/mol) were more than 2-fold greater compared to curcumin (368.385 g/mol) and piperine (285.343 g/mol). From the greater number of atoms in the bigger ligands, it was initially assumed that they were able to form more interactions that could contribute to greater stabilization with the receptor. However, in this case, since the CAS binding site is located at the base of a ~20-Å-deep and ~5-Å-narrow gorge (Cheng et al., 2017), it is believed that the limited space within the binding cavity was unable to accommodate the larger structures of bacoside A and chebulinic acid, causing the ligands to adopt unfavorable conformations, hence the poorer score. Another postulation for bacoside A and chebulinic acid to have higher binding energies can be due to their abilities to bind and interact with either CAS or PAS at a time instead of simultaneously as seen in the interactions of curcumin and piperine towards AChE. For reference, known AChE inhibitors such as donepezil and physostigmine (used in the treatment of myasthenia gravis) with smaller MW of 379.500 g/mol and 375.352 g/mol, respectively, have a similar range of MW to curcumin and piperine, thereby reaffirming our rationale of correlating molecular size to binding space.

From the identification of SER203, GLU334, and HIS447 as the key amino acids involved in the hydrolysis of ACh, it is postulated that access of ACh to the catalytic triad would be blocked if ligands were bound to any of these residues. Hence, the potential of enzymatic inhibition in ligands can be evaluated based on the evidence of SER203, GLU334, and/or HIS447 interaction(s). As for PAS, contacts with TYR72, ASP74, TYR124, TRP286, and/or TYR341 can be deemed as successful inhibition of PAS. From Table 1, only curcumin and piperine achieved contact with both sites while bacoside A and chebulinic acid interacted with PAS only.

The orientation adopted by each ligand within the AChE gorge can be elucidated based on the interaction with amino acids. As mentioned above, the three CAS amino acids are buried deep inside the gorge while five amino acids forming the PAS are located close to the entrance of the gorge (Johnson et al., 2003). From Figure 3, the successful interactions with both sites as seen in curcumin and piperine were due to their linear structures, enabling the molecules to span the length of the gorge. In contrast, the evidence of only PAS interaction seen in bacoside A and chebulinic acid indicates that the structures were not able to penetrate deeply into the CAS. This is especially evident in bacoside A, where the bulky glycoside group was unable to cross the bottleneck gorge entrance. Although several bacoside A and chebulinic acid with higher binding energies were seen to interact with CAS amino acids, this was achieved at the expense of adopting conformations that caused further penalties to the docking affinities (lower-ranked pose results not shown). Since a potential inhibitor has to be able to competitively bind with AChE in the presence of ACh (Nair and Hunter, 2004), only the top pose is relevant and considered in this study.

In addition, insights into the bottleneck structure at the AChE gorge entrance have shown that even endogenous ACh substrate could only cross the narrowest portion when the gate opens momentarily (Henchman et al., 2002; Cheng et al., 2017). In light of this transient entry mechanism, we presume that an inhibitor ligand with dual-site binding capability would be a more potent and efficacious drug compared to ligands that bind to either site only, by reasoning that the former would only need to cross the gate once. This, therefore, underscores the importance of selecting inhibitor ligands that are both CAS and PAS binders (Fang et al., 2014) and, at the same time, forms stable ligand-receptor complex with AChE. Hence, the drug potential of the four ligands is determined based on binding energies and evidence of interaction with the key amino acids at both sites.

In molecular surface studies, it is observed that CAS and PAS of AChE consist of mainly hydrogen bond donors. Therefore, it will form a greater number of hydrogen bonds with ligands rich in hydrogen bond acceptors because both are required in order to form hydrogen bonding (Brown, 2009). Although it was postulated that chebulinic acid with greater number of hydrogen bond acceptors should be able to bind to AChE with the greatest affinity, it is ranked the third in terms of binding energy to AChE. The penalty in score imposed by the presence of unfavorable bonds is most likely attributed to bulkiness of chebulinic acid structure as mentioned above. This analysis further validates the reasoning behind our docking results. The second part of molecular surface interaction studying hydrophobicity gives a preliminary idea on the penetrance ability of compounds across the blood-brain barrier. Theoretically, high hydrophobicity gives higher log *P*-values that indicate how effective a ligand can enter the central nervous system and bind to AChE in the brain. Since bacoside A has the largest area of hydrophobicity, it can be suggested that it has the highest log *P*-value. To confirm this postulation, however, further analysis such as quantitative structure-activity relationship (QSAR) has to be conducted to determine the actual hydrophobicity of ligands.

In sum, from the *in silico* results, it is suggested that curcumin and piperine with good affinities to target sites have met the two criteria set out in this study, namely, as an inhibitor of ACh hydrolysis through CAS interaction and, concurrently, preventing Aβ plaque aggregation through PAS inhibition. On the other hand, bacoside A and chebulinic acid may not be suitable drug candidates for AD. Thus, only curcumin and piperine were selected for further analyses for its AChE inhibition activity and biochemical assay *via* *in vitro* methods.

For *in vitro* studies, these two selected plant-based compounds were tested on their own and in combination with each other. This co-effect approach that is gaining traction was employed on the grounds that synergistic effects of plant combinations exhibit augmented functional properties compared to the effects of individual plants alone (Pereira et al., 2014). Encouraging data reported in combined administration of curcumin and piperine in studies relating to benzo(a)pyrene toxicity (Sehgal et al., 2012) and diethylnitrosamine-induced hepatocellular carcinoma (Patial et al., 2015) have provided further evidence that combination treatment using these two compounds may provide more pronounced results than on their own. As such, in recent years, synergy evaluation has become a key area in phytomedicine research exploring the underlying scientific principles for their therapeutic advantages of multidrug combinations over single constituents often observed in traditional medicine (Wagner and Ulrich-Merzenich, 2009).

For *in vitro* AChE enzyme screening, donepezil was used as a positive control and its percentage inhibition values, as well as its IC_50_ value, were compared to curcumin and piperine. It was noted that donepezil inhibited AChE in a dose-dependent manner and reached its maximal inhibition between 100 and 250 μg/ml. As for the test compounds, the percentage of inhibition for the combination showed that they were much more effective than using curcumin and piperine singularly. Although investigation into the AChE inhibitory potential of these compounds have been done, no study has tested the compounds in combination. Since our data of curcumin and piperine, singularly, are consistent with other published results, the greater inhibition values obtained in this study, when both compounds were used in combination, is indeed promising. For reference, in regard to piperine alone, Kumar et al. (2012) discovered that piperine had a highly significant inhibitory effect on AChE in both aqueous and ethanolic extract. Likewise, the study by Ingkaninan et al. (2003) on Thai traditional and neurotonic remedial plants also showed that *Piper nigrum* extracts at 0.1 mg/ml containing piperine had a 50%–65% inhibitory activity on AChE. As for curcumin alone, Ahmed and Gilani (2009) found that curcumin showed the least potency when compared to other curcuminoids present in turmeric. In the case for dementia, Murata et al. (2015) showed that black pepper and turmeric extracts were potent compounds in inhibiting AChE, proposing that both extracts could have preventive and therapeutic effects on dementia.

As for the combination of curcumin and piperine, we observed in our data that almost half of the concentration of piperine was required to give the desired combinatory effects with curcumin, which again explained the enhancing nature of piperine (Singh et al., 2015). In light of accumulating evidences of AChE accelerating Aβ deposition, which indicate that AChE could play a role in the early stages of senile plaque development (Inestrosa et al., 1996), the promising results of curcumin and piperine in *in vitro* enzyme screening corroborates with our *in silico* predictions that the inhibitory interactions with PAS of AChE may prevent Aβ aggregation. A previous study has shown that AChE has secondary non-cholinergic functions including the processing and deposition of Aβ (Castro and Martinez, 2006). Moreover, AChE co-localizes with Aβ in neuritic plaques and accelerates the assembly of Aβ into fibrils. In turn, the Aβ peptide regulates AChE expression, assembly and glycosylation in cell culture, and transgenic mice and brain of AD patients (Hu et al., 2003). Thus, inhibition of the Aβ aggregation process within the AChE is believed to be beneficial in preventing AD progression.

In regard to compound concentrations, combinatory studies of curcumin and piperine in different *in vivo* studies show that piperine, at all doses used in the studies, can enhance the serum concentration, extent of absorption, and bioavailability of curcumin up to 2000% in both rats and humans with no adverse effects (Shoba et al., 1998). Thus, this allays the toxicity concern even if curcumin and piperine may be needed to be consumed in larger amounts for its therapeutic effects. In sum, these findings further substantiate the claims that curcumin and piperine may be developed as alternative synthetic AChE inhibitors for preventative purposes in AD.

With the growing burden of AD care and increasingly younger age of AD patients, we recognize the need of a compound that has therapeutic roles in addition to preventative functions. Hence, we extended this study by evaluating the effect of curcumin and piperine on Aβ toxicity *via* an *in vitro* cell line. To determine whether the compounds can inhibit Aβ42-induced cytotoxicity, we examined the effect of Aβ42 added to the cell medium upon dissolution and Aβ42 pre-incubated with all test compounds (donepezil, curcumin, and piperine used singularly and in combination) in SH-SY5Y cells (Figure 9). The optimized concentration of combined curcumin and piperine was selected for subsequent analyses in this study. We observed that Aβ exerted degeneration effects toward the cells, as indicated by a drop in cell viability, and the effect was reversed after treatment with all test compounds. More significantly, cell viability was enhanced in Aβ-treated SH-SY5Y cells upon pre-treatment with combined curcumin and piperine as compared to singular compounds. This suggested that combined compounds exhibit greater protective effect in cells when exposed to cytotoxic Aβ. On its own, protecting neuron cells from Aβ neurotoxicity by treating them with curcumin have also been shown in other studies. A recent article focused on the potential effect of curcumin upon Aβ-induced cytotoxicity in HT-22 cells showed a remarkable increase in cell viability, following 48 h of treatment with curcumin, when compared to cells that were treated with Aβ1–42 only (Zhang et al., 2018). Likewise, another study revealed that curcumin at lower doses (0.1, 0.5, and 2.5 μM) enhanced the proliferation of neural stem cells, but at higher doses (12.5 and 62.5 μM), it reduced neural stem cell proliferation (Xiaoxiao et al., 2015), which was also concordant with our findings. Neuroprotective effects of individual curcumin (Yin et al., 2012) and piperine (Chonpathompikunlert et al., 2011) on cells have also been reported. While this study is the first *in vitro* investigation into combinatory curcumin and piperine, a parallel finding can be drawn from Chonpathompikunlert et al. (2011) who reported that piperine coupled with nitroxide radical-containing nanoparticles (RNPs) can increase the viability of SH-SY5Y cells that have been induced with 20 μM Aβ1–42. Their results also showed that a viability of almost 100% in SH-SY5Y was achieved when piperine was used in the presence of 1 mM RNP. However, in the case of piperine used singularly, the viability was decreased at high piperine concentration region (>1 mM) due to its cytotoxicity. This finding is consistent with our results whereby EC of individual piperine was obtained at a lower concentration of 25 μM (Figure 7).

To account for the significant inhibition by compound on Aβ42-induced cell toxicity, we carried out further experiments designed to elucidate the effects of test compounds upon aggregation process of Aβ42 using ThT fluorescence assay. Our results showed that all test compounds were able to inhibit fibrillation of Aβ42 (Figure 10A) as well as to disaggregate the fibrils (Figure 10B), as displayed in the lower intensity of the ThT fluorescence. ThT is a well-known marker for protein fibril formation as the fluorescence increases upon binding to fibril aggregates composed of beta strands. The toxicity of Aβ peptides correlates well with their propensity to aggregate. The process of fibrillogenesis begins with amyloid monomers that assemble to form dimers and small oligomer species, followed by aggregation of oligomers to further construct short, irregular protofibrils and elongate into insoluble fibrils *via* a complex multistep-nucleated polymerization (Cheignon et al., 2018). Once Aβ aggregates extracellularly to form fibrils, it becomes resistant to proteolytic cleavage (Durell et al., 2018). Our strategy was to either inhibit or disaggregate the fibril formation process to prevent amyloidogenesis by using optimized concentrations of singular and combined compounds.

In the last decade, considerable progress has been made on using curcumin in AD therapy. Several lines of evidence suggested that curcumin has anti-amyloid properties in AD. Findings from an *in vivo* study revealed that curcumin inhibits Aβ aggregation as well as disaggregates to form fibrillar Aβ40 (Yang et al., 2005). A previous study also reported that curcumin-functionalized gold nanoparticles (Au-curcumin) of hydrodynamic diameter 10–25 nm was able to inhibit amyloid fibrillation and disintegrate amyloid fibrils (Palmal et al., 2014). In their research, they showed that curcumin was water-soluble when in nanoparticle form, which made it efficient to interact with amyloid proteins, thus offering an enhanced ability in inhibiting amyloid fibrillation and dissolving amyloid fibrils. Furthermore, curcumin and its derivatives were reported to inhibit the fibrillar Aβ formation from Aβ monomer and destabilizes preformed fibrillar Aβ *in vitro* as well, indicating that curcumin was protective against Aβ toxicity (Ono et al., 2003), which is consistent with our findings. While emerging evidence on curcumin against Aβ has been reported, involvement of piperine in Aβ fibrillation process has yet to be elucidated. But from our data, piperine might play a role in disintegrating the Aβ fibril formation. Recent studies reported that indirect involvement of piperine-coated gold formation was able to inhibit amyloid-prone residues of insulin (Anand et al., 2017, 2018). They found that the process of both spontaneous and seed-induced (misfolded protein aggregates) amyloid formation of insulin was strongly inhibited in the presence of piperine-coated gold nanoparticles. Furthermore, the piperine molecule has a unique structure, which is known to interact with proteins and DNA molecules through viable H-bonds mediated *via* its C = O and –O– functional groups (Chinta et al., 2015; Yeggoni et al., 2015). Formation of strong H-bonds between piperine and valine residues of protein molecules has already been reported (Zsila et al., 2005). These evidence might suggest the mechanism of piperine interaction with Aβ, which we would need to further validate in the future.

Several studies have shown that Aβ can produce oxidative stress (Kim et al., 2003; Cutler et al., 2004; Chauhan and Chauhan, 2006). Upon deposition in the brain, Aβ induces oxidative changes that render nerve cell vulnerable to insults. Aβ-mediated oxidative stress could be due to either the increase in ROS production, decrease in enzyme activities involved in the antioxidant defense system, or dysfunctional mitochondria (Chauhan and Chauhan, 2006). In order to evaluate the deleterious effect caused by Aβ and the reverse effect contributed by natural compounds, we analyzed the oxidative product and the activity of antioxidant enzymes in terms of Aβ damage. We observed that our findings were in agreement with previous findings mentioned above where Aβ caused substantial toxic effects towards the antioxidant defense mechanism, as indicated by the significant increase in MDA level and reduced levels of glutathione and catalase enzymes. However, upon treatment with all test compounds, a reversal of the deleterious effects caused by Aβ was noted. More remarkably, a more pronounced rehabilitation effect was observed for the combination of curcumin and piperine as compared to individual compounds.

Cell membrane is made up of phospholipids that are composed of polyunsaturated fatty acids, which serve as the prime targets of ROS. The presence of a double bond adjacent to the methylene group makes the methylene C–H bond of polyunsaturated fatty acids weaker, and therefore, the hydrogen becomes more prone to elimination. Production of free radicals such as hydroxyl radical (•OH), alkoxy radicals (RO•), and peroxy radicals (ROO•) results in the initiation of the lipid peroxidation sequence. Several studies suggested that Aβ enhances oxidative stress by increasing lipid peroxidation. The Aβ25–35 (Aβ containing hydrophilic and hydrophobic domains) treatment enhanced lipid peroxidation as arbitrated by the intensified levels of thiobarbituric-acid-reactive substances in the brain (Um et al., 2006). These products of lipid oxidation structurally modify proteins by covalent interactions and inhibition of enzyme function. Since lipids are membrane components, the interaction of Aβ aggregates with membrane results in impairment of cellular processes, causing oxidative stress and increased free calcium ion concentration that eventually leads to apoptotic cell death (Kirkitadze and Kowalska, 2005). GPx and catalase are the two main enzymes involved in cellular protection against damages due to oxygen-derived free radicals. In other words, these are important scavenging enzymes that can reduce H_2_O_2_ (Flohé and Günzler, 1984; Chelikani et al., 2004), which suggests a role in AD pathology. Altered glutathione metabolism in association with increased oxidative stress has been implicated in the pathogenesis of AD (Reid and Jahoor, 2001). Decreased GSH in lymphoblasts carrying presenilin and APP gene mutations indicate the role of presenilin and APP in oxidative stress in AD (Cecchi et al., 1999). Based on these literature findings, the role of natural products in preventing oxidative stress has been extensively studied (Kim et al., 2005; Calaf et al., 2011; Carocho and Ferreira, 2013; Ali et al., 2018; Fischer et al., 2018; Simioni et al., 2018).

Our findings are in agreement with other studies on the antioxidant effect of curcumin against oxidative damages. A previous study reported that curcumin enhanced the levels of glutathione, superoxide dismutase, and catalase in the brains of lead-poisoned rats while it significantly reduced lead-induced damage (Shukla et al., 2003). Curcumin also protects PC12 cells and normal human umbilical endothelial cells from Aβ-induced oxidative stress (Park and Kim, 2002). Moreover, using the same SY5Y cells as was selected in this study, Uğuz et al. (2016) demonstrated that curcumin can suppress the levels of lipid peroxidation, glutathione, and caspase that were induced by peroxides. In addition, according to Calaf et al. (2011), curcumin is a good scavenger of H_2_O_2_ at high concentrations (over 27 μM) and inhibition of peroxidation by curcuminoids is primarily attributed to the scavenging of the reactive free radicals involved in such a process. As for piperine, it has also been used against oxidative stress in that it plays a crucial role in reducing chemical carcinogen-induced oxidative stress (Khajuria et al., 1998). Piperine enhances the action of phase II detoxification enzyme and reduces the level of DNA damage and DNA–protein cross-links (Selvendiran et al., 2005). A recent study by Verma et al. (2018) using various biomarkers such as comet and lipid peroxidation revealed that piperine at 35 and 50 mM concentrations can significantly reduce the tail moment and peroxidation of lipids induced by cadmium (Cd), which further acknowledges the protective effects of piperine. Hence, it can be concluded that curcumin and piperine have potentiated these scavenging enzymes with different pathways to decrease oxidative stress, thereby possibly serving as a therapeutic tool for AD.

## Conclusion

Curcumin, piperine, bacoside A, and chebulinic acid are plant-based compounds with long-rooted beliefs of cognitive benefits that have been extensively used in food and traditional medicine. This study investigated the potential of these compounds as preventative and therapeutic drugs for AD. Divided into two parts, the *in silico* section has narrowed down the compounds to only curcumin and piperine, based on their favorable binding affinities to AChE and simultaneous interactions with key amino acids of CAS and PAS. On the other hand, bacoside A and chebulinic acid may not be suitable drug candidates owing to their inabilities to adopt favorable conformations within the narrow AChE gorge binding sites.

The second part of this study through *in vitro* methods have demonstrated that curcumin combined with piperine is able to protect SH-SY5Y cells against Aβ-induced cytotoxicity, fibrillation, and oxidative damage by attenuating the toxic effects on neuron cells, inhibiting and disaggregating the fibrils as well as suppressing the generation of ROS. To date, this article is the only study that focuses on the combinatory effect of curcumin and piperine for AD treatment. Although many studies have been conducted on these two compounds, none has adopted the synergistic approach by using individual compound effects as the baseline comparison. We believe that the consistency of our results for individual compounds with other publications substantiates the methodology used for our combinatory studies. In addition, the strength of this study is supported by the corroboration of results between *in silico* prediction and *in vitro* testing on AChE inhibition. On the whole, this report reinforces the benefits of co-effects from different compounds in treating challenging diseases such as AD.

The effects might also involve amyloidogenic and antioxidant properties in different pathways of curcumin and piperine, as well as interactions between complex AChE and Aβ, which requires further investigation. Furthermore, synergistic behavior indicates that the combination of these two compounds at lower concentration may provide a better outcome rather than treatment with singular compounds. Further experiments at a molecular level and animal studies are currently underway.

## Data Availability

The raw data supporting the conclusions of this manuscript will be made available by the authors, without undue reservation, to any qualified researcher.

## Author Contributions

PM and AC contributed to the conception and design of the study. AAM, AT and WL organized the database, ran the experiments and drafted the manuscript. AAM, AT, WL and SV performed the statistical analysis. PM, AC and SV wrote sub-sections of the manuscript. All other authors contributed to manuscript revision, proofread and approved the submitted version.

## Conflict of Interest Statement

The authors declare that the research was conducted in the absence of any commercial or financial relationships that could be construed as a potential conflict of interest.
