# Supplementary material for: Synergistic Effects of Curcumin and Piperine as Potent Acetylcholine and Amyloidogenic Inhibitors With Significant Neuroprotective Activity in SH-SY5Y Cells via Computational Molecular Modeling and in vitro Assay
Source: Front Aging Neurosci. 2019 Aug 27;11:206. doi: 10.3389/fnagi.2019.00206 (PMC6718453; doi:10.3389/fnagi.2019.00206)
Supplement: Supplementary file 1 [file Table_1.DOCX]

Supplementary Material

# Supplementary Table

**Table A.** List of Interactions between Ligands and AChE.

| Ligand | Interactions | | Distance (Å) | Category | Types |
| --- | --- | --- | --- | --- | --- |
|  | From Chemistry | To Chemistry |  |  |  |
| Curcumin | H atom of GLY122 | O atom of curcumin | 2.611 | Hydrogen Bond | Conventional Hydrogen Bond |
|  | H atom of TYR124 | O atom of curcumin | 2.437 | Hydrogen Bond | Conventional Hydrogen Bond |
|  | H atom of SER203 | O atom of curcumin | 2.154 | Hydrogen Bond | Conventional Hydrogen Bond |
|  | H atom of SER203 | O atom of curcumin | 2.508 | Hydrogen Bond | Conventional Hydrogen Bond |
|  | H atom of PHE295 | O atom of curcumin | 1.935 | Hydrogen Bond | Conventional Hydrogen Bond |
|  | TRP286 | Curcumin | 4.033 | Hydrophobic | Pi-Pi Stacked |
|  | Curcumin | TRP286 | 3.724 | Hydrophobic | Pi-Pi Stacked |
| Piperine | H atom of GLY121 | O atom of piperine | 2.322 | Hydrogen Bond | Conventional Hydrogen Bond |
|  | H atom of GLY122 | O atom of piperine | 2.224 | Hydrogen Bond | Conventional Hydrogen Bond |
|  | H atom of SER203 | O atom of piperine | 2.280 | Hydrogen Bond | Conventional Hydrogen Bond |
|  | H atom of PHE295 | O atom of piperine | 2.169 | Hydrogen Bond | Conventional Hydrogen Bond |
|  | C atom of piperine | O atom of GLU202 | 3.596 | Hydrogen Bond | Carbon Hydrogen Bond |
|  | C atom of piperine | O atom of SER 293 | 3.268 | Hydrogen Bond | Carbon Hydrogen Bond |
|  | Piperine | TRP86 | 3.766 | Hydrophobic | Pi-Sigma |
|  | TRP286 | Piperine | 5.076 | Hydrophobic | Pi-Pi Stacked |
|  | TRP86 | Piperine | 4.561 | Hydrophobic | Pi-Alkyl |
|  | TYR337 | Piperine | 5.451 | Hydrophobic | Pi-Alkyl |
|  | HIS447 | Piperine | 5.106 | Hydrophobic | Pi-Alkyl |
| Bacoside A | H atom of bacoside A | O atom of bacoside A | 2.284 | Hydrogen Bond | Conventional Hydrogen Bond |
|  | H atom of bacoside A | O atom of bacoside A | 2.050 | Hydrogen Bond | Conventional Hydrogen Bond |
|  | H atom of bacoside A | O atom of bacoside A | 2.446 | Hydrogen Bond | Conventional Hydrogen Bond |
|  | Bacoside A | TRP286 | 3.937 | Hydrophobic | Pi-Sigma |
|  | Bacoside A | LEU289 | 5.369 | Hydrophobic | Alkyl |
|  | TYR124 | Bacoside A | 5.305 | Hydrophobic | Pi-Alkyl |
|  | TRP286 | Bacoside A | 5.273 | Hydrophobic | Pi-Alkyl |
|  | TRP286 | Bacoside A | 4.474 | Hydrophobic | Pi-Alkyl |
|  | TRP286 | Bacoside A | 5.245 | Hydrophobic | Pi-Alkyl |
|  | TRP286 | Bacoside A | 4.849 | Hydrophobic | Pi-Alkyl |
|  | TRP286 | Bacoside A | 4.563 | Hydrophobic | Pi-Alkyl |
|  | HIS287 | Bacoside A | 4.443 | Hydrophobic | Pi-Alkyl |
|  | PHE297 | Bacoside A | 4.405 | Hydrophobic | Pi-Alkyl |
|  | TYR337 | Bacoside A | 5.084 | Hydrophobic | Pi-Alkyl |
|  | PHE338 | Bacoside A | 5.182 | Hydrophobic | Pi-Alkyl |
|  | TRP286 | Bacoside A | 2.345 | Unfavorable | Unfavorable Bump |
|  | TRP286 | Bacoside A | 2.337 | Unfavorable | Unfavorable Bump |
|  | TYR341 | Bacoside A | 1.958 | Unfavorable | Unfavorable Bump |
|  | Bacoside A | Bacoside A | 2.120 | Unfavorable | Unfavorable Bump |
|  | Bacoside A | Bacoside A | 1.727 | Unfavorable | Unfavorable Bump |
|  | Bacoside A | Bacoside A | 1.578 | Unfavorable | Unfavorable Bump |
|  | Bacoside A | Bacoside A | 1.045 | Unfavorable | Unfavorable Bump |
|  | ARG296 | Bacoside A | 2.669 | Unfavorable | Unfavorable Donor-Donor |
| Chebulinic acid | H atom of chebulinic acid | O atom of ARG296 | 2.047 | Hydrogen Bond | Conventional Hydrogen Bond |
|  | H atom of chebulinic acid | O atom of ARG296 | 1.797 | Hydrogen Bond | Conventional Hydrogen Bond |
|  | H atom of chebulinic acid | O atom of TYR72 | 2.463 | Hydrogen Bond | Conventional Hydrogen Bond |
|  | H atom of SER293 | O atom of chebulinic acid | 2.401 | Hydrogen Bond | Conventional Hydrogen Bond |
|  | H atom of TYR337 | O atom of chebulinic acid | 2.317 | Hydrogen Bond | Conventional Hydrogen Bond |
|  | Chebulinic acid | TRP286 | 4.858 | Electrostatic | Pi-Cation |
|  | ASP74 | Chebulinic acid | 4.068 | Electrostatic | Pi-Anion |
|  | Chebulinic acid | HIS287 | 2.614 | Hydrogen Bond | Pi-Donor Hydrogen Bond |
|  | HIS287 | Chebulinic acid | 5.214 | Hydrophobic | Pi-Pi Stacked |
|  | Chebulinic acid | TYR124 | 5.217 | Hydrophobic | Pi-Pi T-shaped |
|  | TRP286 | Chebulinic acid | 4.797 | Hydrophobic | Pi-Pi T-shaped |
|  | Chebulinic acid | LEU76 | 4.912 | Hydrophobic | Pi-Alkyl |
|  | TRP286 | Chebulinic acid | 5.078 | Hydrophobic | Pi-Alkyl |
|  | TRP286 | Chebulinic acid | 5.198 | Hydrophobic | Pi-Alkyl |
|  | Chebulinic acid | ARG296 | 1.011 | Unfavorable | Unfavorable Donor-Donor |
